# Challenge or Opportunity? Impacts of Falls among Older Adults Living with Dementia on their Care Partners

**DOI:** 10.1093/geroni/igab046.3367

**Published:** 2021-12-17

**Authors:** Yuanjin Zhou, Emily Ishado, Tatiana Sadak

**Affiliations:** 1 The University of Texas at Austin, Austin, Texas, United States; 2 University of Washington, Seattle, Seattle, Washington, United States

## Abstract

Previous studies suggest that falls among community-dwelling older adults living with dementia (OLWD) harm the health and wellbeing of their family/friend care partners. However, little is known about the process through which falls impact care partners. We conducted a grounded theory analysis using 59 semi-structured interviews with care partners of OLWD who were recently hospitalized and had a history of falls. We identified several areas of care partners’ functioning that were affected by falls in positive and negative ways: everyday life, health management for OLWD, and interactions with healthcare providers. Both the fall events and fall risks had negative consequences of reducing care partners' self-care activities and work productivity. Other adverse consequences of fall risks were (1) care partners’ fatigue and conflicts with OLWD due to the intense requirement of daily monitoring, and (2) hesitance to ask healthcare providers for assistance because clinicians frequently did not teach care partners how to address fall risks and might recommend institutionalization. However, OLWD's fall events became a transition point for some care partners to seek support and gain more information and skills about managing OLWD’s health conditions, which might reduce care partners’ burden in the long term. Because OLWD’s falls may have negative and positive consequences for care partners, both problem-solving and strength-based fall management approaches are needed. These strategies focus on developing and sustaining care partners’ self-care, developing collaborative relationships with OLWD, enhancing successful capacity for OLWD’s health management, and cultivating partnerships with healthcare providers.

